# Comparison of different software for processing physical activity measurements with accelerometry

**DOI:** 10.1038/s41598-023-29872-7

**Published:** 2023-02-18

**Authors:** Sanne Verhoog, Cédric Gubelmann, Arjola Bano, Taulant Muka, Oscar H. Franco, Pedro Marques-Vidal

**Affiliations:** 1 Institute of Social and Preventive Medicine, University of Bern, Bern, Switzerland; 2 Department of Medicine, Internal Medicine, Lausanne University Hospital (CHUV) and University of Lausanne, Office BH10-642, Rue du Bugnon 46, 1011 Lausanne, Switzerland; 3 Department of Cardiology, Inselspital, Bern University Hospital, University of Bern, Bern, Switzerland

**Keywords:** Cardiovascular diseases, Lifestyle modification, Preventive medicine

## Abstract

Several raw-data processing software for accelerometer-measured physical activity (PA) exist, but whether results agree has not been assessed. We examined the agreement between three different software for raw accelerometer data, and associated their results with cardiovascular risk. A cross-sectional analysis conducted between 2014 and 2017 in 2693 adults (53.4% female, 45–86 years) living in Lausanne, Switzerland was used. Participants wore the wrist-worn GENEActive accelerometer for 14 days. Data was processed with the GENEActiv manufacturer software, the Pampro package in Python and the GGIR package in R. For the latter, two sets of thresholds “White” and “MRC” defining levels of PA and two versions (1.5–9 and 1.11–1) for the “MRC” threshold were used. Cardiovascular risk was assessed using the SCORE risk score. Time spent (mins/day) in stationary, light, moderate and vigorous PA ranged from 633 (GGIR-MRC) to 1147 (Pampro); 93 (GGIR-White) to 196 (GGIR-MRC); 19 (GGIR-White) to 161 (GENEActiv) and 1 (GENEActiv) to 26 (Pampro), respectively. Spearman correlations between results ranged between 0.317 and 0.995, while concordance coefficients ranged between 0.035 and 0.968. With some exceptions, the line of perfect agreement was not in the 95% confidence interval of the Bland–Altman plots. Compliance to PA guidelines varied considerably: 99.8%, 98.7%, 76.3%, 72.6% and 50.2% for Pampro, GENEActiv, GGIR-MRC v.1.11–1, GGIR-MRC v.1.4–9 and GGIR-White, respectively. Cardiovascular risk decreased with increasing time spent in PA across most software packages. We found large differences in PA estimation between software and thresholds used, which makes comparability between studies challenging.

## Introduction

Accelerometers are a valuable tool for objective measurement of the duration and intensity of physical activity (PA), and accelerometer use in epidemiological research has increased in recent decades^1–3^. With the advent of the accelerometer, PA measurement has greatly improved^4^. However, until today, there is no consensus on standardized methods to collect, process and analyse accelerometer data^4^, and many different accelerometers from different companies are available on the market.

In the past, most studies relied on accelerometer data processed and analysed by the software package provided by the accelerometer manufacturer. Recently, several open access software able to process raw data have been developed. These software could theoretically allow a better standardization and comparability between studies. However, the quantification of stationary behaviour and light, moderate and vigorous PA relies on specific thresholds, and the software package used. Despite several proposed thresholds in the literature based on calibration studies^5–7^, there is no agreement regarding which thresholds to apply for each specific software. Indeed, the wide range of analytical approaches allows shaping the outcome of a study by the choice of a specific PA analysis method and threshold^4^.

Moderate and vigorous PA is inversely associated with cardiovascular disease (CVD), and recommendations regarding the minimum amount of PA to prevent CVD have been issued before^8,9^. Hence, an adequate evaluation of PA is important to assess those associations and to monitor PA at the individual level, which is challenging because of various ways to analyse accelerometer data. It is not clear whether the association between PA and CVD is the same when different software and thresholds are used for analysis.

Comparisons between different types and brands of accelerometers^10–12^, and between thresholds to define PA^13,14^ have been performed. Still, the studies assessing thresholds were conducted in children^13^ or used uniaxial accelerometers^14^, and both used counts to assess PA. Further, to our knowledge, no studies have been performed comparing the different raw-data processing software and their corresponding thresholds, using the same accelerometer device. Additionally, no studies have investigated whether the results of different software processing accelerometer-measured PA can be compared reliably. This could further impact the comparability of results from previous studies on the association of PA on CVD outcomes.

The first aim of this study was to examine the agreement between three different commonly used software packages for processing raw data from the GENEActiv accelerometer: the GENEActiv manufacturer standard software, the Pampro package in Python and the GGIR package in R. For the GGIR package, we further examined two different sets of thresholds. The second aim was to examine the difference in strength of association between PA and health outcomes depending on the software used.

## Materials and methods

### Study population

The study was carried out within the CoLaus Study. Detailed description of the recruitment and follow-up procedures of the CoLaus Study has been described previously^15^. Briefly, the CoLaus Study is a population-based cohort exploring the biological, genetic, and environmental determinants of cardiovascular disease. A non-stratified, representative sample of the population of Lausanne (Switzerland) was recruited between 2003 and 2006 based on the following inclusion criteria: (i) age 35–75 years and (ii) willingness to participate. The second follow-up occurred 10.9 years after the baseline survey and included an optional module assessing the participants’ PA for 14 days with an accelerometer. Hence, data of the second follow-up was used for this study. Overall, 4881 subjects participated in this follow-up.

### Inclusion and exclusion criteria

For the first aim, participants were excluded if they did not participate in the accelerometry or had an insufficient number of valid days for assessment (less than 5 weekdays or 2 weekend days). For the second aim, participants were further excluded if they were aged over 65 years, had a history of myocardial infarction, stroke, or diabetes mellitus. Supplementary Fig. 1 provides details of the participants excluded at each step.

### Physical activity assessment

Accelerometer PA was assessed using a wrist-worn triaxal accelerometer (*GENEActive*, Activinsights Ltd, UK). This device has been validated against reference methods^6^ and has been used in other population studies such as the Fenland^7^ and the Whitehall II^16^ in the UK and the Pelotas^17^ in Brazil. The accelerometers were pre-programmed with a 50 Hz sampling frequency and subsequently attached to the participants’ right wrist. Participants were requested to wear the device continuously (24-h per day) for 14 days in their free-living conditions. Non-wear time was defined by the software based on built-in specific criteria.

Data were analysed according to three different software packages; the original GENEActiv macrocommand file “General physical activity” version 1.9 (GENEActiv, Activinsights Ltd., United Kingdom); the open-access Pampro package^18^, and with R-package GGIR (http://cran.r-project.org)^16,19^. The original macrocommand uses the 60 s-epochized files, while Pampro and GGIR use the “raw” binary files produced by the device. For the GGIR package, two different thresholds were used; one was derived from the Whitehall II study^16^ (referred to as GGIR-White) and the other was identical to the ones used by Pampro (referred to as GGIR-MRC). Finally, two different versions of GGIR were used (v.1.5–9 and v.1.11–1) with the set of thresholds used by Pampro.

Both R-files from the GGIR package are presented in Supplementary Information 1 and 2. For this study, we used the time spent in stationary behaviour (SB), light (LPA), moderate (MPA) and vigorous (VPA) physical activity as provided by the software/thresholds. Table 1 provides an overview of the thresholds for categorization of physical activity for the different software.

**Table 1:** Thresholds for categorization of physical activity for the different software.

	GENEActiv MACRO^6^	Pampro^18^ §	GGIR-White^7^	GGIR-MRC v.1.4–9 *	GGIR-MRC v.4.0.3 *
Thresholds for sedentary	< 241 g.min	< 48 mg	< 85 mg	< 48 mg	< 48 mg
Light	241–338 g.min	48–154 mg	85–181 mg	48–154 mg	48–154 mg
Moderate	339–1131 g.min	154–389 mg	182–436 mg	154–389 mg	154–389 mg
Vigorous	≥ 1132 g.min	≥ 389 mg	≥ 437 mg	≥ 389 mg	≥ 389 mg

^§^https://github.com/Thomite/pampro ; *computed from table S1 of^7^.

Compliance to the WHO guidelines on PA (i.e. 150 min of moderate-to-vigorous intensity PA per week)^9^ was examined for the different software.

### Cardiovascular disease risk

Sex was self-reported. Age at the time of examination was rounded to the nearest year. Smoking was self-reported in a questionnaire and categorized into current smokers and non-smokers (i.e. never and former smokers). Systolic blood pressure was measured with an Omron HEM-907 automated oscillometric sphygmomanometer after at least a 10-min rest in a seated position, and the average of the last two measurements was used. Cholesterol was measured by CHOD-PAP on a Cobas 8000 (Roche Diagnostics, Basel, Switzerland) apparatus, with maximum inter and intra-batch CVs of 1.6%-1.7%.

Cardiovascular disease risk was assessed using the Systematic Coronary Risk Evaluation (SCORE) model as recommended for European countries^8^. This model predicts the ten-year risk of fatal CVD based on age, sex, smoking status, systolic blood pressure, total cholesterol, and HDL cholesterol concentrations; values above 5% are considered as high risk and values above 10% as very high risk^20^. The SCORE model is applicable to individuals aged 45–64 with no previous history of CVD. Therefore, we additionally excluded 1183 individuals aged over 65 years and those with a history of myocardial infarction, stroke or diabetes mellitus for this analysis.

### Statistical analysis

Descriptive results were expressed as number of participants (percentage) or as average ± standard deviation. SB and PA levels, as well as differences of time spent in SB and activity levels according to the different software and thresholds, were expressed in median and interquartile range (IQR). Between-software or between-threshold comparisons were performed using Wilcoxon signed rank test for paired samples.

Spearman correlations were used to associate the different software and thresholds with each other; 95% confidence intervals (CIs) were obtained by bootstrapping with replacement, using 1000 iterations and bias-corrected values. Lin’s concordance correlation coefficient and corresponding 95% CI was used to measure the agreement between the different software and thresholds^21^. Bland–Altman plots were used to visualize the extent of (dis)agreement between the software.

Linear regression analysis was used to associate SB, LPA and MVPA with the SCORE values. All activity levels (SB, LPA, MPA and VPA) were divided into tertiles and added as dummy variables in the regression analysis, whereby the first tertile served as the reference group.

Statistical significance was assessed for two-sided tests with p < 0.05. All statistical analyses were performed using Stata version 15.0 for windows (Stata Corp, College Station, Texas, USA).

### Ethical approval

The institutional Ethics Committee of the University of Lausanne, which afterwards became the Ethics Commission of Canton Vaud (www.cer-vd.ch) approved the baseline CoLaus study (reference 16/03, decisions of 13th January and 10th February 2003). The approval was renewed for the first (reference 33/09, decision of 23rd February 2009) and the second (reference 26/14, decision of 11th March 2014) follow-ups. The full decisions of the CER-VD can be obtained from the authors upon request. The study was performed in agreement with the Helsinki declaration and its former amendments, and in accordance with the applicable Swiss legislation. All participants gave their signed informed consent before entering the study.

## Results

### Characteristics of participants

Of the initial 4881 participants, 2693 (53.4% female, age range 45–86 years) were considered eligible for analysis (Supplementary Information 3, Fig. 1). The characteristics of included and excluded participants are presented in Supplementary Information 4 1. Included participants were younger and less likely to be female.

### Differences between the software

Table 2 presents the time spent in SB and each activity level according to the different software and thresholds in time expressed as minutes per day, or as a proportion of time expressed as percentages. Time spent in stationary, light, moderate and vigorous PA ranged from 609 (GGIR-MRC version 1.11–1) to 1147 (Pampro); 93 (GGIR-White) to 211 (GGIR-MRC version 1.11–1); 19 (GGIR-White) to 161 (GENEActiv) and 1 (GENEActiv and GGIR-White) to 26 (Pampro) mins/day, respectively. The findings for the different levels of PA were similar when PA was expressed as percentage of time. All differences were significant at *p* < 0.001.

**Table 2:** Descriptive statistics for stationary behaviour and physical activity levels according to the GENEACTIV manufacturer, the PAMPRO package, the GGIR package with two different thresholds (White and MRC) and two versions of the GGIR package (1.5–9 and 1.11–1) for the MRC threshold. CoLaus study, Lausanne, Switzerland, 2014–2017, on 2693 participants.

	GENEACTIV	PAMPRO	GGIR-White	GGIR-MRC, v. 1.5–9	GGIR-MRC, v. 1.11–1
As time (min/day)					
SB	647 [567; 722]	1147 [1101;1192]	742 [701; 787]	633 [580; 681]	609 [554; 662]
Light PA	108 [85; 132]	177 [152; 201]	93 [67; 125]	196 [150; 245]	211 [166; 261]
Moderate PA	161 [114; 223]	88 [72; 106]	19 [11; 32]	31 [18; 47]	34 [20; 51]
Vigorous PA	1 [0; 5]	26 [19; 35]	1 [0; 3.]	2 [1; 4]	2 [1; 5]
As % of time					
SB	70.3 [62.1; 77.1]	79.7 [76.5; 82.8]	86.6 [81.9; 90.5]	73.5 [66.7; 79.2]	73.5 [66.7; 79.2]
Light PA	11.5 [9.3; 13.9]	12.3 [10.6; 13.9]	10.6 [7.8; 14.2]	22.5 [17.7; 27.4]	24.5 [19.7; 29.6]
Moderate PA	17.2 [12.4; 23.8]	6.1 [5.0; 7.4]	2.2 [1.3; 3.6]	3.5 [2.1; 5.4]	3.8 [2.4; 5.8]
Vigorous PA	0.1 [0; 0.5]	1.8 [1.3; 2.4]	0.1 [0.1; 0.3]	0.2 [0.1; 0.5]	0.2 [0.1; 0.5]

*SB* stationary behaviour; *PA* physical activity. Results are expressed as median and interquartile range. Between-group comparisons performed using Wilcoxon signed-rank test; all differences are significant at *p* < 0.001. Percentages do not add to 100 due to rounding and differences in distribution.

Table 3 presents the differences for time spent in SB and each activity level between the different software and thresholds expressed as mins/day. Compared to GENEActiv, which is the standard software from the device manufacturer, Pampro overestimated SB, LPA, and VPA, and underestimated MPA. The GGIR-White software overestimated SB and underestimated LPA and MPA. The GGIR-MRC version 1.5–9 software overestimated LPA, and underestimated SB and MPA. The GGIR-MRC version 1.5–9 or 1.11–1 software overestimated LPA, and underestimated SB and MPA. These findings were similar for PA expressed as percentage of time (Table 4).

**Table 3:** Differences for stationary behaviour and physical activity levels in absolute time between the different software, CoLaus study, Lausanne, Switzerland, 2014–2017, on 2693 participants.

	Stationary behaviour	Light physical activity	Moderate physical activity	Vigorous physical activity
GENEACTIV vs				
PAMPRO	− 499 [− 555; − 443]	− 66 [− 80; − 53]	74 [41; 119]	− 22 [− 29; − 17]
GGIR-White	− 101 [− 157; − 44]	15 [− 9; 36]	141 [100; 192]	0 [0; 2]
GGIR-MRC, v.1.5–9	15 [− 28; 57]	− 83 [− 116; − 57]	130 [91; 177]	0 [− 1; 1]
GGIR-MRC, v.1.11–1	38 [1; 75]	− 99 [− 131; − 73]	128 [90; 174]	− 1 [− 1; 0]
PAMPRO vs				
GGIR-White	403 [352; 452]	78 [60; 96]	66 [55; 78]	24 [17; 31]
GGIR-MRC, v.1.5–9	518 [478; 557]	− 20 [− 50; 7]	54 [44; 64]	23 [17; 30]
GGIR-MRC, v.1.11–1	542 [500; 582]	− 36 [− 67; − 9]	52 [42; 61]	23 [17; 30]
GGIR-White vs				
GGIR-MRC, v.1.5–9	− 114 [− 138; − 89]	− 100 [− 122; − 78]	− 11 [− 16; − 7]	− 1 [− 1; 0]
GGIR-MRC, v.1.11–1	138 [106; 170]	− 116 [− 140; − 93]	− 13 [− 20; − 8]	− 1 [− 2; 0]
GGIR-MRC, v.1.5–9 vs				
GGIR-MRC, v.1.11–1	23 [4; 42]	− 16 [− 24; − 7]	− 2 [− 5; 0]	0 [0; 0]

SB, stationary behaviour; PA, physical activity. Results are expressed as median and [interquartile range]. Positive/negative values indicate that the software indicated in bold overestimates/underestimates relative to the other. Between-group comparisons performed using Wilcoxon signed-rank test; all differences are significant at *p* < 0.001.

**Table 4:** Differences for stationary behaviour and physical activity levels as percentage of time between the different software, CoLaus study, Lausanne, Switzerland, 2014–2017, on 2693 participants.

	Stationary behaviour	Light physical activity	Moderate physical activity	Vigorous physical activity
GENEACTIV vs				
PAMPRO	− 9.6 [− 15.0; − 5.2]	− 0.7 [− 2.1; 0.9]	11.2 [7.2; 16.4]	− 1.4 [− 1.9; − 1.0]
GGIR-White	− 16.1 [− 20.2; − 12.6]	0.8 [− 1.8; 3.3]	14.9 [10.8; 20.3]	0 [− 0.1; 0.1]
GGIR-MRC, v.1.5–9	− 3.1 [− 5.4; − 1.1]	− 10.5 [− 13.9; − 7.5]	13.6 [9.8; 18.6]	0 [− 0.1; 0]
GGIR-MRC, v.1.11–1	− 0.9 [− 2.5; 0.7]	− 12.4 [− 15.9; − 9.4]	13.3 [9.5; 18.2]	− 0.1 [− 0.1; 0]
PAMPRO vs				
GGIR-White	− 6.4 [− 8.5; − 4.2]	1.3 [− 1.0; 3.4]	3.6 [2.8; 4.4]	1.5 [1.1; 2.0]
GGIR-MRC, v.1.5–9	6.4 [3.1; 10.1]	− 10.2 [− 13.9; − 6.8]	2.4 [1.4; 3.2]	1.4 [1.1; 1.9]
GGIR-MRC, v.1.11–1	8.9 [5.2; 12.8]	− 12.2 [− 16.1; − 8.6]	2.2 [1.0; 3.0]	1.4 [1.0; 1.9]
GGIR-White vs				
GGIR-MRC, v.1.5–9	13.0 [10.5; 15.6]	− 11.5 [− 13.8; − 9.2]	− 1.3 [− 1.8; − 0.8]	− 0.1 [− 0.1; 0]
GGIR-MRC, v.1.11–1	15.4 [12.5; 18.2]	− 13.5 [− 16.0; − 11.0]	− 1.5 [− 2.3; − 1.0]	− 0.1 [− 0.2; 0]
GGIR-MRC, v.1.5–9 vs				
GGIR-MRC, v.1.11–1	2.2 [1.1; 3.4]	− 1.9 [− 2.9; − 1.0]	− 0.2 [− 0.5; 0]	0 [− 0.1; 0]

*SB* stationary behaviour; *PA* physical activity. Results are expressed as median and [interquartile range]. Positive/negative values indicate that the software indicated in bold overestimates/underestimates relative to the other. Between-group comparisons performed using Wilcoxon signed-rank test; all differences are significant at *p* < 0.001.

### Agreement between software and thresholds

Table 5 presents Spearman correlations and Lin’s concordance coefficients between the software and thresholds for SB and all levels of PA. The correlations and concordances were slightly better when PA was expressed as percentage of time. All correlations between both GGIR thresholds were high. When comparing the three different software (GENEActiv, Pampro and GGIR) and GGIR versions with activity expressed as mins/day, the correlations for SB ranged from 0.317 (Pampro with GGIR-White) to 0.918 (GGIR-MRC version 1.5–9 and version 1.11–1). For VPA the correlations ranged from 0.700 (GENEActiv with Pampro) to 0.995 (GGIR-White and GGIR-MRC version 1.5–9). The concordance coefficients for SB ranged from 0.019 (Pampro with GGIR-White) to 0.875 (GGIR-MRC version 1.5–9 and version 1.11–1). For vigorous PA, the concordance coefficients ranged from 0.092 (Pampro with GGIR-White) to 0.968 (GGIR-White and GGIR-MRC version 1.5–9). Supplementary Information 3, Fig. 2 depict the associations between the different software for each type of PA.

**Table 5:** Spearman correlations and Lin’s concordance coefficients between the different software/thresholds, for the different physical activity components, CoLaus study, Lausanne, Switzerland, 2014–2017, on 2693 participants.

	As time (minutes/day)	As percentage of time		
	Spearman correlation	Lin concordance	Spearman correlation	Lin concordance
Stationary behaviour				
GENEACTIV and PAMPRO	0.588 (0.556 ; 0.616)	0.035 (0.032 ; 0.037)	0.883 (0.872 ; 0.893)	0.370 (0.356 ; 0.383)
GENEACTIV and GGIR (White)	0.621 (0.596 ; 0.647)	0.339 (0.320 ; 0.358)	0.906 (0.895 ; 0.914)	0.308 (0.296 ; 0.321)
GENEACTIV and GGIR (MRC, v.1.5–9)	0.763 (0.739 ; 0.786)	0.689 (0.672 ; 0.706)	0.946 (0.939 ; 0.953)	0.876 (0.868 ; 0.883)
GENEACTIV and GGIR (MRC, v.1.11–1)	0.821 (0.802 ; 0.838)	0.725 (0.710 ; 0.740)	0.971 (0.965 ; 0.976)	0.947 (0.944 ; 0.950)
PAMPRO and GGIR (White)	0.317 (0.282 ; 0.349)	0.019 (0.017 ; 0.022)	0.844 (0.831 ; 0.858)	0.520 (0.503 ; 0.536)
PAMPRO and GGIR (MRC, v.1.5–9)	0.643 (0.616 ; 0.666)	0.026 (0.024 ; 0.028)	0.916 (0.908 ; 0.923)	0.523 (0.509 ; 0.537)
PAMPRO and GGIR (MRC, v.1.11–1)	0.647 (0.620 ; 0.669)	0.025 (0.024 ; 0.027)	0.903 (0.895 ; 0.912)	0.419 (0.405 ; 0.433)
GGIR (White) and GGIR (MRC, v.1.5–9)	0.874 (0.863 ; 0.884)	0.402 (0.388 ; 0.417)	0.942 (0.937 ; 0.947)	0.397 (0.383 ; 0.410)
GGIR (White) and GGIR (MRC, v.1.11–1)	0.778 (0.760 ; 0.795)	0.288 (0.274 ; 0.302)	0.927 (0.920 ; 0.934)	0.325 (0.313 ; 0.338)
GGIR (MRC v.1.5–9) and (MRC, v.1.11–1)	0.918 (0.910 ; 0.926)	0.875 (0.866 ; 0.883)	0.977 (0.974 ; 0.979)	0.950 (0.947 ; 0.953)
Light physical activity				
GENEACTIV and PAMPRO	0.802 (0.785 ; 0.817)	0.279 (0.265 ; 0.292)	0.727 (0.706 ; 0.746)	0.628 (0.609 ; 0.648)
GENEACTIV and GGIR (White)	0.557 (0.526 ; 0.584)	0.453 (0.425 ; 0.481)	0.496 (0.466 ; 0.527)	0.409 (0.380 ; 0.439)
GENEACTIV and GGIR (MRC, v.1.5–9)	0.778 (0.759 ; 0.795)	0.257 (0.243 ; 0.270)	0.746 (0.727 ; 0.767)	0.195 (0.184 ; 0.206)
GENEACTIV and GGIR (MRC, v.1.11–1)	0.787 (0.769 ; 0.805)	0.214 (0.203 ; 0.226)	0.760 (0.740 ; 0.780)	0.160 (0.151 ; 0.170)
PAMPRO and GGIR (White)	0.737 (0.717 ; 0.758)	0.251 (0.238 ; 0.264)	0.696 (0.673 ; 0.718)	0.535 (0.518 ; 0.553)
PAMPRO and GGIR (MRC, v.1.5–9)	0.875 (0.864 ; 0.885)	0.641 (0.628 ; 0.654)	0.844 (0.831 ; 0.856)	0.173 (0.165 ; 0.182)
PAMPRO and GGIR (MRC, v.1.11–1)	0.869 (0.858 ; 0.880)	0.549 (0.535 ; 0.564)	0.825 (0.810 ; 0.838)	0.135 (0.128 ; 0.142)
GGIR (White) and GGIR (MRC, v.1.5–9)	0.918 (0.912 ; 0.925)	0.341 (0.328 ; 0.354)	0.907 (0.899 ; 0.915)	0.309 (0.297 ; 0.322)
GGIR (White) and GGIR (MRC, v.1.11–1)	0.904 (0.895 ; 0.911)	0.281 (0.270 ; 0.293)	0.891 (0.882 ; 0.901)	0.249 (0.238 ; 0.260)
GGIR (MRC v.1.5–9) and (MRC, v.1.11–1)	0.977 (0.975 ; 0.980)	0.953 (0.950 ; 0.957)	0.976 (0.973 ; 0.978)	0.942 (0.938 ; 0.945)
Moderate physical activity				
GENEACTIV and PAMPRO	0.936 (0.929 ; 0.942)	0.280 (0.270 ; 0.291)	0.905 (0.896 ; 0.912)	0.128 (0.122 ; 0.134)
GENEACTIV and GGIR (White)	0.844 (0.830 ; 0.857)	0.084 (0.079 ; 0.088)	0.826 (0.811 ; 0.842)	0.085 (0.080 ; 0.090)
GENEACTIV and GGIR (MRC, v.1.5–9)	0.876 (0.864 ; 0.886)	0.137 (0.130 ; 0.143)	0.860 (0.848 ; 0.873)	0.139 (0.132 ; 0.146)
GENEACTIV and GGIR (MRC, v.1.11–1)	0.884 (0.874 ; 0.895)	0.147 (0.139 ; 0.154)	0.874 (0.863 ; 0.886)	0.153 (0.145 ; 0.160)
PAMPRO and GGIR (White)	0.783 (0.765 ; 0.800)	0.135 (0.128 ; 0.143)	0.761 (0.742 ; 0.778)	0.274 (0.261 ; 0.288)
PAMPRO and GGIR (MRC, v.1.5–9)	0.821 (0.806 ; 0.834)	0.253 (0.241 ; 0.265)	0.798 (0.782 ; 0.814)	0.519 (0.502 ; 0.537)
PAMPRO and GGIR (MRC, v.1.11–1)	0.817 (0.801 ; 0.831)	0.278 (0.265 ; 0.291)	0.793 (0.777 ; 0.810)	0.564 (0.546 ; 0.581)
GGIR (White) and GGIR (MRC, v.1.5–9)	0.989 (0.988 ; 0.990)	0.806 (0.798 ; 0.815)	0.988 (0.987 ; 0.989)	0.799 (0.790 ; 0.808)
GGIR (White) and GGIR (MRC, v.1.11–1)	0.973 (0.969 ; 0.976)	0.744 (0.733 ; 0.754)	0.971 (0.967 ; 0.975)	0.729 (0.718 ; 0.740)
GGIR (MRC v.1.5–9) and (MRC, v.1.11–1)	0.982 (0.979 ; 0.985)	0.977 (0.976 ; 0.979)	0.981 (0.978 ; 0.984)	0.974 (0.972 ; 0.976)
Vigorous physical activity				
GENEACTIV and PAMPRO	0.700 (0.678 ; 0.719)	0.152 (0.142 ; 0.162)	0.694 (0.672 ; 0.715)	0.238 (0.223 ; 0.252)
GENEACTIV and GGIR (White)	0.835 (0.820 ; 0.848)	0.794 (0.783 ; 0.804)	0.819 (0.802 ; 0.834)	0.816 (0.805 ; 0.826)
GENEACTIV and GGIR (MRC, v.1.5–9)	0.835 (0.820 ; 0.849)	0.853 (0.844 ; 0.862)	0.831 (0.815 ; 0.845)	0.867 (0.859 ; 0.876)
GENEACTIV and GGIR (MRC, v.1.11–1)	0.856 (0.842 ; 0.868)	0.880 (0.872 ; 0.887)	0.848 (0.835 ; 0.863)	0.893 (0.886 ; 0.900)
PAMPRO and GGIR (White)	0.755 (0.736 ; 0.772)	0.092 (0.086 ; 0.099)	0.728 (0.708 ; 0.747)	0.157 (0.146 ; 0.168)
PAMPRO and GGIR (MRC, v.1.5–9)	0.774 (0.756 ; 0.789)	0.121 (0.113 ; 0.129)	0.758 (0.739 ; 0.776)	0.209 (0.196 ; 0.222)
PAMPRO and GGIR (MRC, v.1.11–1)	0.771 (0.752 ; 0.788)	0.128 (0.120 ; 0.136)	0.749 (0.731 ; 0.768)	0.224 (0.211 ; 0.237)
GGIR (White) and GGIR (MRC, v.1.5–9)	0.995 (0.994 ; 0.995)	0.968 (0.966 ; 0.970)	0.959 (0.955 ; 0.963)	0.965 (0.963 ; 0.968)
GGIR (White) and GGIR (MRC, v.1.11–1)	0.967 (0.960 ; 0.972)	0.925 (0.920 ; 0.930)	0.933 (0.924 ; 0.939)	0.920 (0.915 ; 0.925)
GGIR (MRC v.1.5–9) and (MRC, v.1.11–1)	0.972 (0.966 ; 0.977)	0.967 (0.965 ; 0.970)	0.952 (0.945 ; 0.958)	0.964 (0.962 ; 0.967)

Results are expressed as Spearman rank correlation or Lin concordance coefficients and (95% confidence intervals). For Spearman correlation coefficients, 95% confidence intervals were bootstrapped and bias-corrected values are presented. Spearman correlation indicates the association between values, while Lin concordance coefficient evaluates the degree to which pairs of observations fall on the Y0X (i.e. the 45° line). All coefficients are statistically significant at *p* < 0.001.

There was no clear pattern or consistency of correlations and concordances between the software. For example, GENEActiv and Pampro were highly correlated (r = 0.936) for MPA but the correlation was lower (r = 0.588) for SB. Similarly, the concordance between GENEActiv and GGIR-MRC version 1.5–9 was high (0.853) for VPA and low (0.137) for MPA.

The Bland–Altman plots are presented in Supplementary Information 3, Figs. 3  to 12. With some exceptions (e.g. between GENEActiv and Pampro), the line of perfect agreement was not in the 95% CI of the Bland–Altman plots. Additionally, most Bland–Altman plots showed a linear trend and increasing disagreement with increasing time spent in all PA levels.

### Compliance to recommendations and association with 10-year CVD risk

Compliance to the WHO recommendations on PA varied widely between the software and thresholds: 99.8%, 98.7%, 72.6%, 76.3% and 50.2% for Pampro, GENEActiv, GGIR-MRC v.1.4–9, GGIR-MRC v.1.11–1 and GGIR-White, respectively.

**Figure 1:**
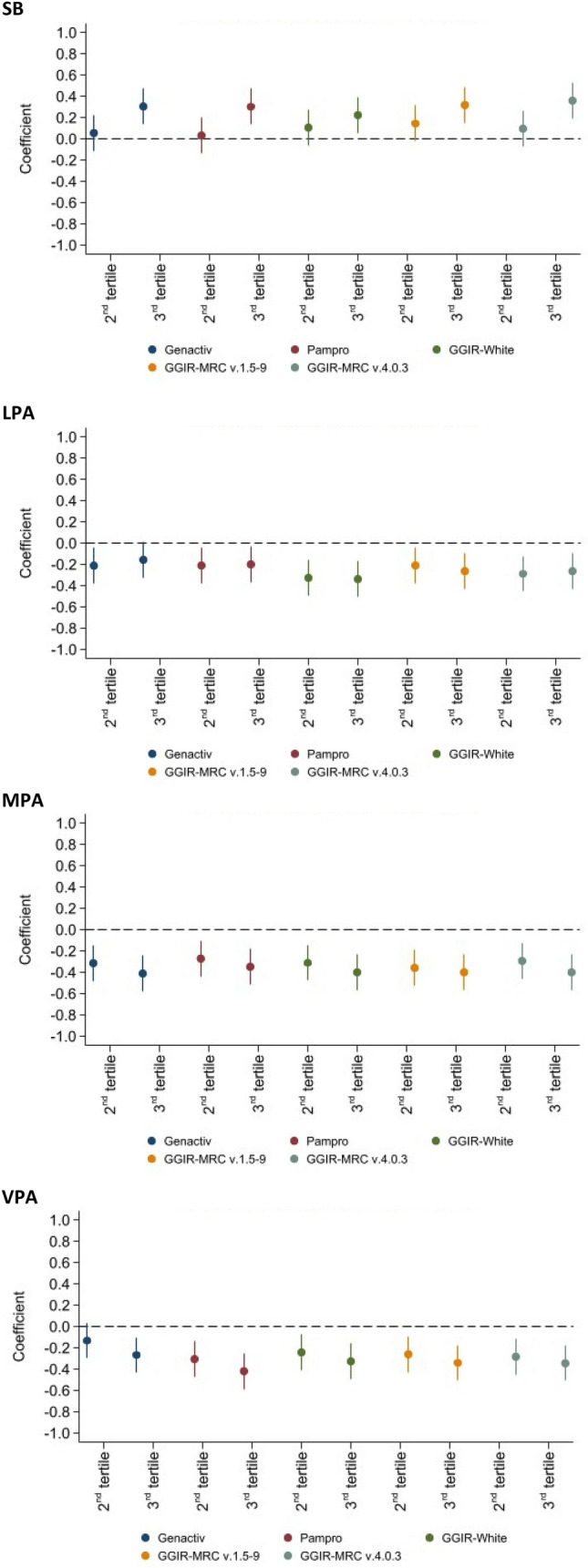
Association of stationary behaviour (SE), light (LPA), moderate (MPA), and vigorous (VPA) physical activity with cardiovascular risk as assessed using SCORE for different software, CoLaus study, Lausanne, Switzerland, 2014–2017. Y-axis shows cardiovascular disease risk represented by SCORE. X-axis represents tertiles of time spent in the corresponding PA level, whereby the first tertile served as reference.

The association between CVD risk and tertiles of time spent in SB, LPA, MPA and VPA according to the different software are summarized in Fig. 1. Overall, increased time spent in SB was associated with an increased CVD risk, while increased time spent in VPA was associated with a decreased risk. Still, the magnitude of the associations varied by software and threshold. For example, only the highest tertile of VPA according to GENEActiv was associated with a significant decrease in CVD risk, while both the middle and highest tertile of VPA according to the other software/thresholds were associated with a significant decrease in CVD risk.

## Discussion

We found large differences in PA estimation between different software and thresholds applied. Those differences resulted in discrepancies regarding important metrics for public health, such as the prevalence of compliance to PA guidelines, or the association with cardiovascular disease (CVD) risk.

### Agreement between software and thresholds

With a few exceptions, Spearman correlations were good and Lin concordance coefficients were poor, the latter information suggesting that values from one software/threshold pair either overestimate or underestimate those obtained using another software/threshold pair. Therefore, it is not possible to derive the results of one software from another, and comparison between studies is difficult, as no simple converting method can be applied. There was no consistent pattern regarding the differences between the software. The Bland–Altman plots showed that in most cases the line of perfect agreement was not in the 95% confidence interval, indicating a systematic disagreement between the two software and/or thresholds considered. Additionally, the disagreement between software and/or thresholds increased with increasing time spent in all levels of PA.

Accelerometers are useful tools to objectively assess PA in large-scale studies, but their utilization requires standardization. Many authors have called for harmonization of data collection, processing criteria and selection of cut-points to assess PA, so to allow comparability between studies^22–24^. Our results show that despite using the same data collection and processing criteria, the use of different software and thresholds leads to discrepant estimates of time spent in different intensities of PA. Interestingly, compared to the GENEActiv software, all open access software underestimated MPA and overestimated VPA. Time spent in SB was considerably higher for PAMPRO than for the other packages. A likely explanation is that is that PAMPRO does not differentiate between sleep and sedentary behavior, while the other packages do and provide information on sleep duration^25^.

Although the performance of the different software has been tested and validated in controlled laboratory settings, their validation in a “real world” setting has seldom been performed. The GENEActiv software has been validated using a shaker and 60 adults aged 40 to 65 performing different activity tasks in a laboratory^6^. The GGIR software has been validated in a large study (*N* = 4094, age range 60–83) regarding the assessment of sleep^26^, but we failed to find validation studies regarding PA levels. The Pampro package has been recently validated against doubly labelled water in a sample of 193 subjects aged 40–66^27^. Similarly, the assessment of the thresholds to define SB, LPA, MPA and VPA relied on proxy measurements such as heart rate and movement sensors^7^. Overall, our results suggest that most software and/or thresholds have been validated for people aged over 40, using different techniques. Joint validation of the different software and thresholds using reference methods is urgently needed.

### Compliance to recommendations and association with 10-year CVD risk

The lack of agreement between software and/or thresholds resulted in a wide variation in the prevalence of subjects compliant with the WHO recommendations for PA. This finding is in agreement with a previous study conducted in children^28^, where the prevalence of children meeting the recommended 60 min/day of MVPA ranged between 8 and 96% depending on the threshold used. Among healthy subjects, a UK study using wrist-worn accelerometers and data processed by GGIR obtained an average time spent in MVPA > 100 min/day^29^, while the corresponding values in a US study using waist-worn accelerometers was < 40 min/day^30^. Notably, the values for the US study were twenty minutes less than the average time spent in MVPA by subjects with diabetes and cardiovascular disease of the UK study^29^. Overall, our results suggest that prevalence of (non) compliance to PA recommendations cannot be reliably compared between studies using different software to analyse PA data and/or thresholds to define light, moderate and vigorous PA.

Across all software used, increased time in SB was associated with a higher CVD risk, while increased time in VPA was associated with a lower CVD risk. These findings are in agreement with recent systematic reviews assessing the association of leisure time PA^31^ and SB^32^ with CVD. However, depending on the software, the strength of the association differed across all levels of PA. This implicates that using different software can lead to different results when examining the association between PA and health outcomes, and results across studies are therefore not comparable.

### Implications for research

Our findings highlight the importance of using common software and thresholds if prevalence of physically active people or associations between PA and disease are to be made. Based on our findings, it was not possible to indicate the most accurate software / threshold, although the Pampro package appeared to be more related with CVD risk than the others are. It is also important that researchers can access the raw acceleration signal rather than the manufacturer-specific data. All these steps would greatly facilitate comparison between studies and ultimately joint (meta) analysis of the data. Alternatively, researchers should make the code / thresholds / software used in their analyses available so that other researchers can apply them to their data^33^. Future studies need to further investigate the potential differences deriving from the different versions of the software used to analyse the data. In addition, the software packages assessing PA levels should be validated against a golden standard, such as doubly labelled water or direct calorimetry, with different age groups. Still, current golden standard methods such as doubly labelled water provide total daily energy consumption, but fail to provide any information regarding intensity and bout duration. Other methods such as oxygen consumption using portable devices could be envisaged, but they remain rather cumbersome and are difficult to use on a free-living, 24-h scale. The surrogate or “silver” methods such as heart rate measurement or activity logs are not sufficient.

### Strengths and limitations

To the best of our knowledge, this is the first study to assess differences in PA estimation with different software and thresholds for processing accelerometer data. Our study comprised a large sample size of 2693 individuals from a well-characterized population-based cohort. Furthermore, we included the full range of activity intensity from SB to VPA, and we assessed the association of different software with a CVD risk score.

This study also has some limitations. The major limitation is that we lacked a “gold standard” that would allow us to assess the accuracy of each software. Furthermore, packages like GGIR or PAMPRO only read in data, apply pre-processing procedures, and then apply algorithms for predicting PA outcomes from features in the signal. In this case, the packages are applying simplistic thresholds based purely on the magnitude of acceleration and ignore other important time and frequency domain features in the accelerometer signal. However, the field is progressing towards the application of machine learning or pattern recognition approaches to overcome this problem^34^. A second limitation is that we included a single population mainly constituted of Caucasian subjects, although the results might not differ if other ethnicities are studied. Furthermore, all assessments were conducted at the same period and whether the variation of levels of PA between software could change with time could not be evaluated.

### Conclusion

We found large differences in PA estimation between software and thresholds, which preclude comparability between studies. Validation of the different software against golden standards is urgently needed. In the meantime, investigators should consider utilizing a single software to facilitate comparison or present results utilizing at least two of the most used software so that findings can be more comparable.

## Supplementary Information

Supplementary Information 1.

Supplementary Information 2.

Supplementary Information 3.

Supplementary Information 4.

## Data Availability

Due to the sensitivity of the data and the lack of consent for online posting, individual data cannot be made accessible. Only metadata will be made available in digital repositories. Metadata requests can also be performed via the study website www.colaus-psycolaus.ch.
