# Supplementary material for: Comparison of different software for processing physical activity measurements with accelerometry
Source: Sci Rep. 2023 Feb 18;13:2879. doi: 10.1038/s41598-023-29872-7 (PMC9938888; doi:10.1038/s41598-023-29872-7)
Supplement: Supplementary file 2 — Supplementary Information 2. [file 41598_2023_29872_MOESM2_ESM.docx]

Appendix 2: R-code to run the GGIR package with MRC thresholds

############################################################################

#Loading packages

# THIS CODE WAS RUN ON VERSIONS V.1.5-9 AND V.1.11-1

# USING MRC THRESHOLDS (https://zenodo.org/record/1187043#.W6uKqIcUmpo)

# https://github.com/Thomite/pampro default thresholds

############################################################################

#install.packages(c("devtools"))

#require(devtools)

#install_version("GGIR", version="1.5-9")

#install_version("GGIR", version="1.11-1")

#install.packages(c ("GGIR", "GENEAread", "zoo", "bitops", "data.table", "mmap"))

ev <- lapply("GGIR", library, character.only=T)

ev

search() # check if all is loaded

mode=c(2,3,4,5)

# Path for the folder where bin files are stored

datadir= "D:/Test/"

#pathname to folder where output should be written to "

# Changed 17.08.2017 to have ndayswindow=14 and maxdur=0

outputdir="D:/Test/"

studyname="COLAUS"

f0 = 1

f1 = c() # f1 = c() for all

g.shell.GGIR (#-------------------------------

# General parameters

#-------------------------------

mode=mode,

datadir=datadir,

outputdir=outputdir,

studyname=studyname,

f0=f0,

f1=f1,

overwrite = TRUE,

do.imp=TRUE,

idloc=1,

print.filename=TRUE,

storefolderstructure = FALSE,

#-------------------------------

# Part 1 parameters:

#-------------------------------

windowsizes = c(5,900,3600),

#deziredtz="Europe/Bern",

do.cal=TRUE,

do.enmo = TRUE,

do.anglez=TRUE,

chunksize=1,

printsummary=TRUE,

#-------------------------------

# Part 2 parameters:

#-------------------------------

strategy = 1,

ndayswindow=14,

hrs.del.start = 1,

hrs.del.end = 1,

maxdur = 15,

includedaycrit = 16,

L5M5window = c(0,24),

M5L5res = 10,

winhr = c(5),

qlevels = c(c(1380/1440),c(1410/1440)),

qwindow=c(0,24),

ilevels = c(seq(0,400,by=50),8000),

mvpathreshold =c(154),

#-------------------------------

# Part 3 parameters:

#-------------------------------

timethreshold= c(5),

anglethreshold=5,

ignorenonwear = TRUE,

#-------------------------------

# Part 4 parameters:

#-------------------------------

excludefirstlast = FALSE,

includenightcrit = 16,

def.noc.sleep = c(21,9),

#loglocation= "pathname to a sleeplog - if using one",

outliers.only = FALSE,

criterror = 4,

relyonsleeplog = FALSE,

sleeplogidnum = TRUE,

colid=1,

coln1=2,

do.visual = FALSE,

nnights = 14,

#-------------------------------

# Part 5 parameters:

#-------------------------------

# Key functions: Merging physical activity with sleep analyses

#threshold.lig = c(30,40,50),

#threshold.mod = c(100,120),

#threshold.vig = c(400,500),

threshold.lig = c(48),

threshold.mod = c(154),

threshold.vig = c(389),

boutcriter = 0.8,

boutcriter.in = 0.9,

boutcriter.lig = 0.8,

boutcriter.mvpa = 0.8,

#boutdur.in = c(10,20,30),

#boutdur.lig = c(1,5,10),

#boutdur.mvpa = c(1,5,10),

boutdur.in = c(10),

boutdur.lig = c(10),

boutdur.mvpa = c(10),

timewindow = c("WW"),

#-----------------------------------

# Report generation

#-------------------------------

do.report=c(5))
