# Supplementary material for: Comparison of different software for processing physical activity measurements with accelerometry
Source: Sci Rep. 2023 Feb 18;13:2879. doi: 10.1038/s41598-023-29872-7 (PMC9938888; doi:10.1038/s41598-023-29872-7)
Supplement: Supplementary file 3 — Supplementary Information 3. [file 41598_2023_29872_MOESM3_ESM.docx]

**Supplementary files**

**Supplementary Table 1.** Characteristics of excluded and included participants, CoLaus study, Lausanne, Switzerland.

|  | **Included (n=2693)** | **Excluded (n=2188)** | **p-value** |
| --- | --- | --- | --- |
| Female (%) | 1439 (53.4) | 1250 (57.1) | < 0.001 |
| Age (years) | 62.0 ± 10.0 | 64.1 ±10.9 | < 0.001 |
| Age groups (%) |  |  | < 0.001 |
| 45-55 | 799 (29.7) | 547 (25.0) |  |
| 55-65 | 855 (31.8) | 647 (29.6) |  |
| 65-75 | 710 (26.4) | 589 (26.9) |  |
| 75+ | 329 (12.2) | 405 (18.5) |  |
| Body mass index (kg/m^2^) | 26.4 ± 4.7 | 26.5 ± 4.8 | 0.55 |
| BMI categories (%) |  |  | 0.13 |
| Underweight | 46 (1.7) | 26 (1.6) |  |
| Normal | 1060 (39.5) | 722 (40.2) |  |
| Overweight | 1094 (40.8) | 681 (37.9) |  |
| Obese | 483 (18.0) | 366 (20.4) |  |

BMI, body mass index. Results are expressed as number of participants (column percentage) or as average ± standard deviation. Between-group comparisons performed using chi-square for categorical variables or student’s t-test for continuous variables. For excluded participants, number of participants does not add to 2188 due to the presence of missing values
