# Supplementary material for: Comparison of different software for processing physical activity measurements with accelerometry
Source: Sci Rep. 2023 Feb 18;13:2879. doi: 10.1038/s41598-023-29872-7 (PMC9938888; doi:10.1038/s41598-023-29872-7)
Supplement: Supplementary file 4 — Supplementary Information 4. [file 41598_2023_29872_MOESM4_ESM.docx]

# Supplementary Figures

**Supplementary Figure 1.** Flowchart for inclusion of participants. Aim 1: comparison between algorithms; aim 2: association with cardiovascular risk.


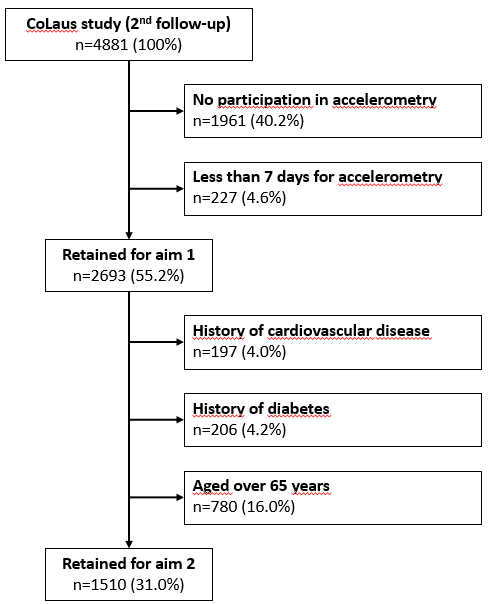


**Supplementary Figure 2.** XY plot for physical activity components (expressed as % of daily time) between the different algorithms, CoLaus study, Lausanne, Switzerland, 2014-2017.

Sedentary behaviour Light physical activity

Moderate physical activity Vigorous physical activity

**Supplementary Figure 3.** Bland-Altman plots between the Geneactiv and the Pampro algorithms, CoLaus study, Lausanne, Switzerland, 2014-2017. Left column: physical activity expressed as min/day; right column: physical activity expressed as percentage of daily time. SE, sedentary; LPA, light PA; MPA, moderate PA; VPA, vigorous PA.

**Supplementary Figure 4.** Bland-Altman plots between the Geneactiv and the GGIR-White algorithms, CoLaus study, Lausanne, Switzerland, 2014-2017. Left column: physical activity expressed as min/day; right column: physical activity expressed as percentage of daily time. SE, sedentary; LPA, light PA; MPA, moderate PA; VPA, vigorous PA.

**** ****

**Supplementary Figure 5.** Bland-Altman plots between the Geneactiv and the GGIR-MRC v.1.5-9 algorithms, CoLaus study, Lausanne, Switzerland, 2014-2017. Left column: physical activity expressed as min/day; right column: physical activity expressed as percentage of daily time. SE, sedentary; LPA, light PA; MPA, moderate PA; VPA, vigorous PA.

**** ****

**Supplementary Figure 6.** Bland-Altman plots between the Geneactiv and the GGIR-MRC v.4.0.3 algorithms, CoLaus study, Lausanne, Switzerland, 2014-2017. Left column: physical activity expressed as min/day; right column: physical activity expressed as percentage of daily time. SE, sedentary; LPA, light PA; MPA, moderate PA; VPA, vigorous PA.

**Supplementary Figure 7.** Bland-Altman plots between the Pampro and the GGIR-White algorithms, CoLaus study, Lausanne, Switzerland, 2014-2017. Left column: physical activity expressed as min/day; right column: physical activity expressed as percentage of daily time. SE, sedentary; LPA, light PA; MPA, moderate PA; VPA, vigorous PA.

**** ****

**Supplementary Figure 8.** Bland-Altman plots between the Pampro and the GGIR-MRC v.1.5-9 algorithms, CoLaus study, Lausanne, Switzerland, 2014-2017. Left column: physical activity expressed as min/day; right column: physical activity expressed as percentage of daily time. SE, sedentary; LPA, light PA; MPA, moderate PA; VPA, vigorous PA.

**** ****

**Supplementary Figure 9.** Bland-Altman plots between the Pampro and the GGIR-MRC v.4.0.3 algorithms, CoLaus study, Lausanne, Switzerland, 2014-2017. Left column: physical activity expressed as min/day; right column: physical activity expressed as percentage of daily time. SE, sedentary; LPA, light PA; MPA, moderate PA; VPA, vigorous PA.

**Supplementary Figure 10.** Bland-Altman plots between the GGR-White and GGIR-MRC v.1.5-9 algorithms, CoLaus study, Lausanne, Switzerland, 2014-2017. Left column: physical activity expressed as min/day; right column: physical activity expressed as percentage of daily time. SE, sedentary; LPA, light PA; MPA, moderate PA; VPA, vigorous PA.

**** ****

**** ****

**** ****

**** ****

**Supplementary Figure 11.** Bland-Altman plots between the GGR-White and GGIR-MRC v.4.0.3 algorithms, CoLaus study, Lausanne, Switzerland, 2014-2017. Left column: physical activity expressed as min/day; right column: physical activity expressed as percentage of daily time. SE, sedentary; LPA, light PA; MPA, moderate PA; VPA, vigorous PA.

**Supplementary Figure 12.** Bland-Altman plots between the GGIR-MRC v.1.5-9 and v.4.0.3 algorithms, CoLaus study, Lausanne, Switzerland, 2014-2017. Left column: physical activity expressed as min/day; right column: physical activity expressed as percentage of daily time. SE, sedentary; LPA, light PA; MPA, moderate PA; VPA, vigorous PA.
